# Interaction between peripheral blood mononuclear cells and *Trypanosoma cruzi*-infected adipocytes: implications for treatment failure and induction of immunomodulatory mechanisms in adipose tissue

**DOI:** 10.3389/fimmu.2024.1280877

**Published:** 2024-03-12

**Authors:** Leyllane Rafael Moreira, Ana Carla Silva, Cíntia Nascimento da Costa-Oliveira, Claudeir Dias da Silva-Júnior, Kamila Kássia dos Santos Oliveira, Diego José Lira Torres, Michelle D. Barros, Michelle Christiane d. S. Rabello, Virginia Maria Barros de Lorena

**Affiliations:** ^1^ Department of Tropical Medicine, Federal University of Pernambuco, Recife, Brazil; ^2^ Department of Immunology, Aggeu Magalhães Institute, Recife, Brazil

**Keywords:** adipose tissue, immunomodulation, *Trypanosoma cruzi*, cytokine, chemokine, adipokine, benznidazole

## Abstract

**Background/Introduction:**

Adipose tissue (AT) has been highlighted as a promising reservoir of infection for viruses, bacteria and parasites. Among them is *Trypanosoma cruzi*, which causes Chagas disease. The recommended treatment for the disease in Brazil is Benznidazole (BZ). However, its efficacy may vary according to the stage of the disease, geographical origin, age, immune background of the host and sensitivity of the strains to the drug. In this context, AT may act as an ally for the parasite survival and persistence in the host and a barrier for BZ action. Therefore, we investigated the immunomodulation of T. cruzi-infected human AT in the presence of peripheral blood mononuclear cells (PBMC) where BZ treatment was added.

**Methods:**

We performed indirect cultivation between T. cruzi-infected adipocytes, PBMC and the addition of BZ. After 72h of treatment, the supernatant was collected for cytokine, chemokine and adipokine assay. Infected adipocytes were removed to quantify T. cruzi DNA, and PBMC were removed for immunophenotyping.

**Results:**

Our findings showed elevated secretion of interleukin (IL)-6, IL-2 and monocyte chemoattractant protein-1 (MCP-1/CCL2) in the AT+PBMC condition compared to the other controls. In contrast, there was a decrease in tumor necrosis factor (TNF) and IL-8/CXCL-8 in the groups with AT. We also found high adipsin secretion in PBMC+AT+T compared to the treated condition (PBMC+AT+T+BZ). Likewise, the expression of CD80+ and HLA-DR+ in CD14+ cells decreased in the presence of T. cruzi.

**Discussion:**

Thus, our findings indicate that AT promotes up-regulation of inflammatory products such as IL-6, IL-2, and MCP-1/CCL2. However, adipogenic inducers may have triggered the downregulation of TNF and IL-8/CXCL8 through the peroxisome proliferator agonist gamma (PPAR-g) or receptor expression. On the other hand, the administration of BZ only managed to reduce inflammation in the microenvironment by decreasing adipsin in the infected culture conditions. Therefore, given the findings, we can see that AT is an ally of the parasite in evading the host‘s immune response and the pharmacological action of BZ.

## Introduction

Chagas disease, caused by the hemoflagellate protozoan *Trypanosoma cruzi*, persists as a significant public health challenge (1). About 6–7 million people worldwide are estimated to be infected with *T. cruzi*, mainly in Latin America, where Chagas disease is considered endemic (2). On the other hand, due to migratory flows, Chagas disease has now spread more widely, and non-endemic areas, such as North America and European countries, have also reported several cases in the recent past (1).

The classic form of Chagas disease transmission is via the vector route, wherein the individual’s damaged skin comes into contact with the contaminated feces of triatomine insects. The lesion allows the entry of trypomastigotes, which differentiate and thus infect and multiply inside the cells. However, other transmission routes also play an important role, such as congenital, transfusion/organ transplant, and oral and laboratory accidents. The onset of infection, which represents the acute phase of Chagas disease, is characterized by high parasitemia and nonspecific symptoms such as fever, malaise, and apathy, often making early diagnosis difficult. Around 2 to 4 months after infection, infected individuals can enter the chronic phase of the disease, which can generate more severe symptomatic manifestations, such as the cardiac, digestive, or mixed form. However, around 70% of patients present an asymptomatic condition called the indeterminate form (3).

Faced with such heterogeneous clinical forms, the immune response associated with each form is quite complex. Patients with the indeterminate form show a more regulatory immune response, with a predominance of anti-inflammatory cytokines such as IL-10. In contrast, the symptomatic clinical forms promote robust inflammation, increasing pro-inflammatory cytokines such as interferon-gamma (IFN-γ) and TNF. Nevertheless, the immunological mechanisms associated with disease progression to asymptomatic or symptomatic forms are not fully elucidated (4). In addition, factors such as comorbidities, the host’s immunological background, or the strain of *T. cruzi* can alter the expected immune response pattern (4, 5).

*T. cruzi* is a versatile parasite with mechanisms for evading the immune response, allowing it to survive and multiply in the infected host (5, 6). Among the evasion strategies, infection of the AT by *T. cruzi* has stood out as a promising reservoir of infection/latency and metabolic modulation of the parasite (7) . The AT is an endocrine organ responsible for the body‘s energy reserve. However, it also has immunological properties, such as the secretion of bioactive factors known as adipokines. AT is divided into three types of tissue: white adipose tissue (WAT), associated with energy storage and secretion of adipokines; brown adipose tissue (BAT), which is associated with thermogenic activity and beige/brite adipose tissue, which comes from WAT, but has thermogenic functions (8, 9).

The visceral AT of mice infected with the Brazil strain of *T. cruzi* shows elevated levels of TNF, IFN-γ and IL-1, as well as reduced adiponectin and leptin, as a reflection of the inflammatory profile of the infection (10). In addition, adipocytes differentiated from murine fibroblasts, upon *in vitro* infection with the Tulahuen strain of *T. cruzi*, demonstrated an inflammatory phenotype, with increased expression of the cytokines IFN-γ, TNF and IL-1β and the chemokines CCL2, CCL5 and CXCL10 (11).

In contrast, administration of murine AT-derived mesenchymal cells in untreated *T. cruzi* Brazil strain-infected mice three days post-infection reduced parasitaemia and inflammation, with increased IL-10 expression through modulation of the immune response (12). However, the immunological mechanisms of differentiated human adipose cells infected with *T. cruzi* and treated with BZ were not verified in such studies. In humans, little is known about the role of this reservoir of infection in Chagas disease; however, the presence of *T. cruzi* kDNA in the AT of patients with the disease has been detected (13). However, the impact of the immune response of this reservoir of infection against the parasite has not yet been explored.

Currently, the drugs available to treat Chagas disease are two nitro heterocyclic compounds: BZ and Nifurtimox. Both drugs have a trypanocidal effect on all evolutionary forms of the parasite (amastigotes, epimastigotes, and trypomastigotes). It is believed that the drugs act on the formation of free radicals and electrophilic metabolites, affecting the parasite’s macromolecules. However, from the 1980s onwards, Nifurtimox was discontinued in Brazil and other South American countries due to its high toxicity compared to BZ and the reduction in its trypanocidal effect in some endemic regions (14–16). Therefore, BZ is the recommended drug in Brazil. However, its efficacy is controversial in the chronic phase of the disease, and some variables such as age, geographical origin and susceptibility of the strains to the drug significantly alter the efficacy of the drug (17, 18).

In this context, once the AT acts as a reservoir of infection, it may represent a barrier to the drug‘s action, thus reducing its effectiveness. In addition, immune response cells such as PBMCs also participate in the immune modulation generated by infection and/or treatment with BZ, which may be an important factor to be evaluated. Therefore, we evaluated the modulation of the immune response induced by human mesenchymal stem cells differentiated into adipocytes infected with *T. cruzi* under indirect contact with PBMC and the addition of BZ treatment to mimic what happens in this microenvironment.

## Materials and methods

### Parasites

Vero cells (ATCC – CCL-81™) cultured in 75 cm^2^ flasks containing RPMI 1640 medium (Sigma™) supplemented with 10% fetal bovine serum (FBS) (GIBCO^®^) and 1% penicillin/streptomycin (Lonza) (complete medium) were used to maintain the parasites. After 70-80% confluence of the cells, trypomastigote forms of *T. cruzi* strain Y (DTU II), kept frozen in liquid nitrogen, were thawed and used to infect the cells. Approximately 1x10^6^ parasites/mL were added to the cell culture for infection in complete medium for 24 hours in an incubator with 5% CO_2_ in a humidified atmosphere. At the end of incubation, the supernatant was discarded to remove the parasites that were unable to infect cells. Complete RPMI 1640 medium (Sigma™) was added, and the cultures were incubated for 5 to 8 days. Vero cells were observed daily under an inverted microscope. After the rupture of the Vero cells, the free trypomastigotes in the culture medium were collected, centrifuged (2555 x g for 10 min at 20°C), and the pellet was stained with Trypan blue (Sigma™) to observe the cell viability of the trypomastigotes that were used in the culture.

### Cultivation of human adipose tissue stem cells (ADSC)

ADSC (PT-5006, LONZA™) were grown in 75 cm³ culture flasks in Dulbecco’s Modified Eagle Medium (DMEM) supplemented with 20% FBS and 1% penicillin-streptomycin. When they reached full confluence, the cells were detached using 2% trypsin/EDTA solution (GIBCO™), washed with complete medium, and seeded in new flasks for further propagation of the cells. All ADSC experiments were performed between the third and sixth cell passages.

### Adipogenic differentiation

When they reached confluence, ADSC were harvested using 2% trypsin/EDTA solution (GIBCO™) and plated at a concentration of 5 x 10^5^ cells/well in individual 35 mm × 10 mm culture plates (CORNING^®^). When they reached about 90% confluence (24/48 hours post-plating), the differentiation wells were cultured with DMEM supplemented with 10% FBS and 1% gentamicin-amphotericin (PT-8205, LONZA™) with the adipogenic inducers (insulin, dexamethasone, 3-isobutyl-methyl-xanthine and indomethacin), according to the recommendations of the adipogenic differentiation kit (PT-9502, LONZA™) for 12 days. A control group of wells, which included cells cultured in DMEM supplemented with 10% FBS and 1% gentamicin-amphotericin, was also included. After the culture time recommended by the manufacturer, the cells were stained with the AdipoRed™ reagent (PT-7009, LONZA™), which uses Nile red dye to highlight lipid vesicles. After adding AdipoRed™ in the wells, the fluorescence of the lipid vesicles was visualized in the 485nm filter under 572nm emission in the confocal microscope.

### Study population

Five volunteer subjects were included, three females and two males, with a mean age of 26.6 ± 4.03. From each participant, 30 mL of blood was collected in vacuum tubes (Vacutainer^®^) containing sodium heparin to obtain PBMC. In addition, 5 mL of blood were collected in dry tubes to obtain serology confirmation of *T. cruzi*. Exclusion criteria for our study were residing in an endemic area for Chagas disease, having received a blood transfusion, being under 18 years of age and presenting reactive serology for Chagas disease or being a carrier of any other chronic inflammatory or autoimmune disease. All study participants answered a research form and signed the Free and Informed Consent Form voluntarily.

### Isolation of peripheral blood mononuclear cells

Blood was dispensed into polypropylene tubes (BD Falcon™) and diluted 1:2 in sterile filtered phosphate-buffered saline (PBS). This solution was added to polypropylene tubes (BD Falcon™) containing Ficoll-Hypaque PLUS™ (Amersham Biosciences) (1:3). Cells were separated by centrifugation for 30 min at 900 x g 20°C, and the PBMC ring formed between the Ficoll interface and plasma was collected. Cells were washed twice by centrifugation and resuspended in DMEM medium (SIGMA™) supplemented with 10% FBS and 1% gentamicin-amphotericin (GIBCO^®^). Cells were counted in a Neubauer chamber using Trypan Blue dye (Sigma™).

### Indirect culture between *T. cruzi* infected adipocytes, PBMC and addition of BZ treatment

**Figure 1:**
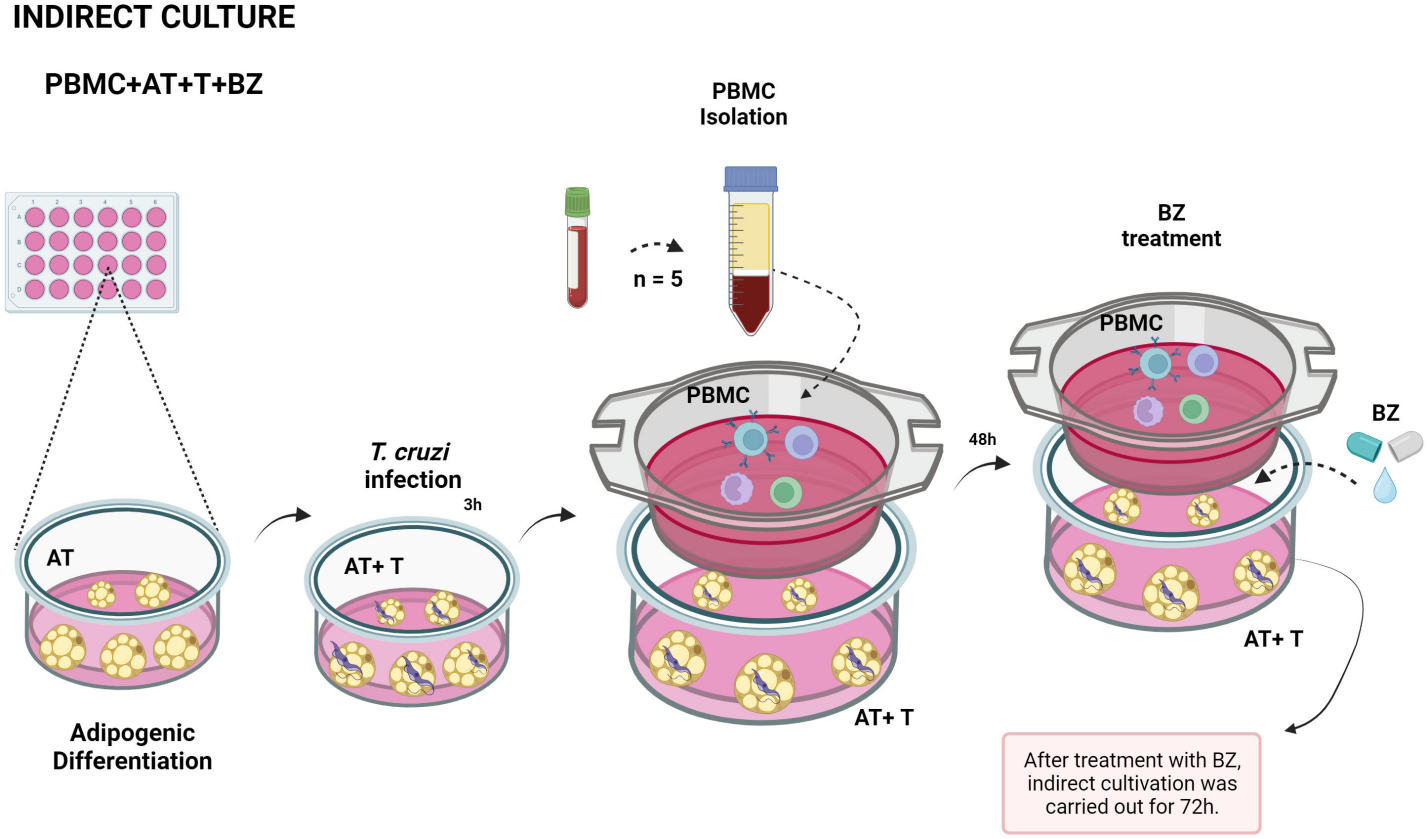
Schematic representation of indirect culture between *Trypanosoma cruzi*-infected AT, PBMC and benznidazole treatment. PBMC, Peripheral blood mononuclear cells (PBMC); AT, Adipose tissue from adipogenic differentiation of ADSC (AT); *Trypanosoma cruzi* (T); Benz nidazole (BZ). Design created with the aid of Biorender ^®^.

Initially, we plated ADSC in 24-well cell culture plates (KASVI) at a concentration of 0.2 x 10^4^ cells/well and performed adipogenic differentiation. After the end of differentiation, we infected these adipocytes with trypomastigote forms of *T. cruzi* strain Y for approximately 3 hours at a ratio of 5:1 parasites per cell as described by Nagajyothi et al. (11). After the infection period, we renewed the culture medium with DMEM supplemented with 10% FBS and 1% gentamicin-amphotericin to remove parasites that had not internalized into the cells. Then, above each cultured well, we added an indirect culture insert with a pore size of 0.4µm (Thincert^®^ - n° 662640 – GREINER) and deposited the PBMC at a concentration of 2 x 10^6^ cells/mL directly on the membrane in DMEM supplemented with 10% FBS and 1% gentamicin-amphotericin and were incubated at 37°C at 5% CO_2_ for 48 h. After this period, we carried out the treatmentwith BZ at a 1µg/mL concentration, according to Romanha et al. (19), and returned the plates to the incubator (**Figure 1**). About 72 h after treatment, according to Moreira et al. (20) we collected the culture supernatant for cytokine and chemokine dosage, removed the PBMC from the inserts for immunophenotyping, and the infected adipocytes for *T. cruzi* DNA quantification.

### Quantification of *T. cruzi-*DNA

After the indirect culture time, cells from each culture condition were removed from the culture plates by washing with ice-cold Phosphate-buffered saline (PBS) and promptly deposited in polypropylene tubes (BD Falcon™ – 15 mL) for washing with PBS (500 x g, 10 min, brake 1). Then, the final pellet was transferred to 1.5 mL microtubes, and extraction was performed with the QIAamp DNA Mini Kit – QIAGEN according to the manufacturer‘s recommendations. The primers TcSAT1 – F (5′AAATTCCTCCAAGCAGCGGA3′) and TcSAT2 – R (5′ ATGAATGGCGGGAGTCAGAG3′) were used to amplify the *T. cruzi* satellite DNA (SAT-DNA), as described previously (21). *T. cruzi* genomic DNA (strain Y) was used to generate a standard curve to quantify the parasite load in samples by quantitative PCR (qPCR). Quantification of parasite load was performed with the QuantStudio 5 Real-Time PCR System (Thermo Fisher Scientific) using the TcSAT-IAM system (21). Samples were tested in duplicate at all stages. The results were analyzed, interpreted, and recorded using the QuantStudio Design and Analysis Software, as described by Moreira et al. (20).

### Cytokine and chemokine assay

After collection of culture supernatants from indirect cultivation, cytokines (IL-2, IL-4, IL-6, IL-10, TNF and IFN-γ) and chemokines (IP-10/CXCL-10, MCP-1/CCL2, MIG/CXCL9, RANTES/CCL5 and IL-8/CXCL8) using CBA (Cytometric Bead Array-BD Biosciences, USA), according to the manufacturer‘s recommendations. Samples were acquired at the Flow Cytometry Technology Platform, located at the Nucleus of Technological Platforms (NPT)/IAM/Fiocruz, through the FACScalibur flow cytometer (Becton Dickson Immunocytometry Systems), with the CellQuestPro software (Beckton Dickson) and analyzed in the FCAP Array 3.1 software.

### Adipokines dosage and acquisition strategy

Adipokines (adiponectin, adipsin, leptin and resistin) were measured using the LEGENDplex™ Human Metabolic Panel 1 kit (Biolegend^®^), according to the manufacturer‘s recommendations. Samples were acquired from the Flow Cytometry Technology Platform, located at NPT/IAM/Fiocruz, using the FACScalibur flow cytometer (Becton Dickson Immunocytometry Systems), with BD CellQuestPro software (Beckton Dickson) and analyzed using LEGENDplex™ Data Analysis software (Biolegend^®^). The adipokine population was selected using pseudocolor density (FSC) versus side scatter (SSC) plots. Eight hundred beads were acquired within window A (Beads A – Adiponectin and Adipsin) and 800 beads from window B (Beads B – Leptin and Resistin). The classification of the beads was performed using the FL4 channel. After selecting the window of interest (A) and (B), the adipokines were analyzed by obtaining two-dimensional plots of the spot fluorescence distribution using LEGENDplex™ Data Analysis (Biolegend^®^) (**Supplementary Figure 1**).

### Immunophenotyping of lymphocytes and monocytes from an indirect culture between *T. cruzi* infected adipocytes, PBMC and addition of BZ treatment

PBMC adhered to the insert were removed using ice-cold PBS-Wash (PBS containing 0.5% bovine serum albumin and 1% sodium azide) and centrifuged at 400 x g for 5 minutes. The supernatant was discarded, and the pellet containing the cells was distributed into cytometry tubes and incubated with monoclonal antibodies anti-CD4/PerCP (BD™), CD8/FITC (BD™), CD28/PE (BD™) and CTLA-4/APC (BD™) for lymphocytes, and anti-CD14/FITC (BD™), CD80/PE (BD™), HLA-DR/PerCP (BD™) for CD14+ cells, for 30 min at room temperature protected from light. After incubation, the cells were washed by centrifugation (400 x g for 5 min at room temperature). Then, the cells were fixed with BD Cytofix™ (BD Biosciences^®^) for 15 min at 4°C. Furthermore, after washing (400 x g for 5 min at room temperature), the cells were resuspended with 300 μL of PBS-Wash/tube and stored at 4°C until the time of acquisition on the FACScalibur flow cytometer (Beckton Dickson). The lymphocyte population was selected FSC versus SSC plots, with 20,000 events acquired within the R1 lymphocyte window, and CD14+ cells were selected via FCS versus FL1, with 1,000 events acquired within the R2 window. After selecting the window of interest (R1) and (R2), CD4+ and CD8+ T lymphocytes and CD14+ cells and molecules were analyzed by obtaining two-dimensional plots of the spot fluorescence distribution using FlowJo^®^ software version 10.8.1.

### Statistical analysis

The results were analyzed using GraphPad PRISM 8.0 Windows^®^ software (USA) and submitted to the Shapiro-Wilk normality test. When the samples showed normal distribution, the One-Way ANOVA test was applied, followed by Tukey’s post-test. However, when they did not follow the normal distribution, we used the Friedman test and Dunn’s post-test to assess the differences between the groups. All conclusions were made at the 5% significance level.

### Ethical considerations

The approaches that were used in the study were approved by the Research Ethics Committee (CEP) of IAM/Fiocruz (C.A.A.E 97930918.3.0000.5190).

## Results

### Adipogenic differentiation of ADSCs and assessment of parasite load of *T. cruzi*-infected adipocytes in indirect culture between AT and PBMC after BZ treatment

**Figure 2:**
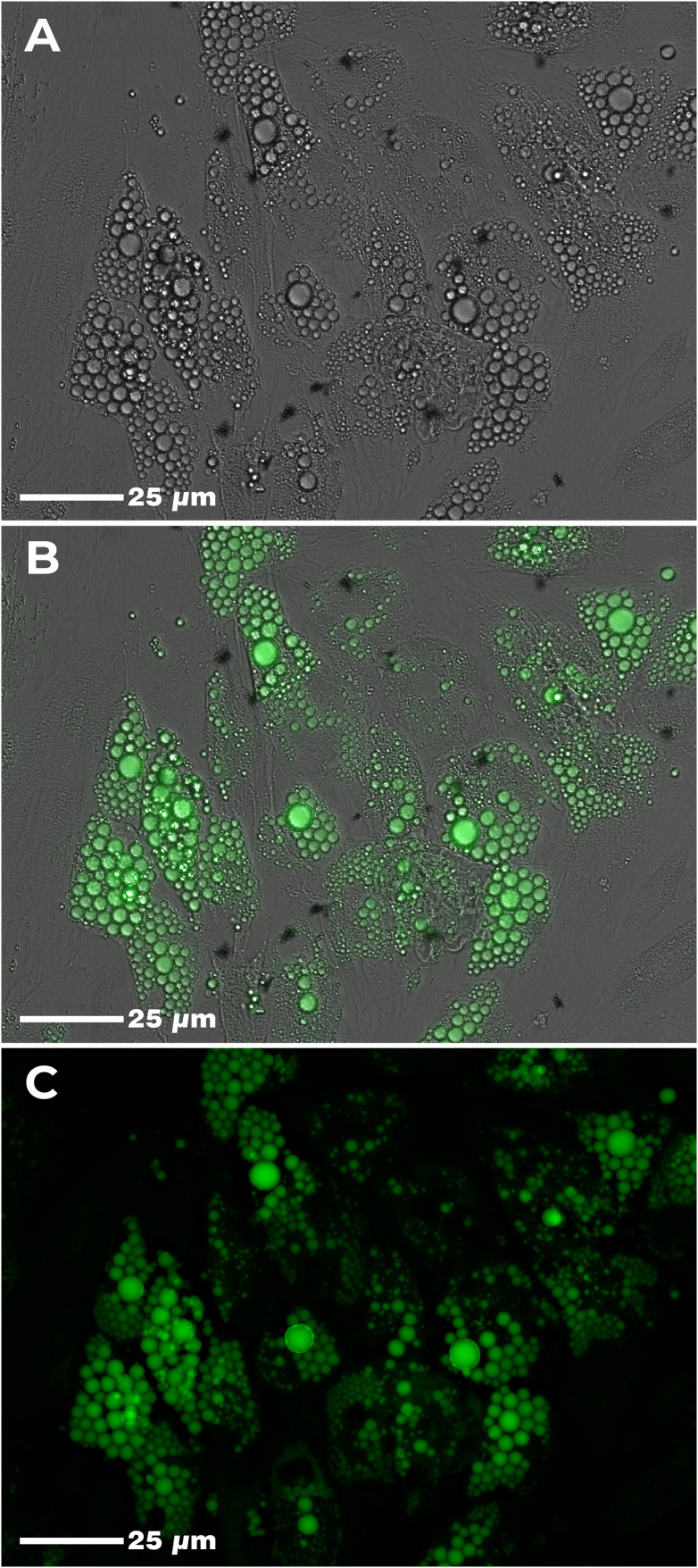
Adipogenic differentiation of human adipose-derived stem cells (ADSC). Legend: Human adipose tissue-derived stem cells (ADSC) after adipogenic differentiation. Lipid vesicles were highlighted by green color after staining with AdipoRed™ and visualized in confocal microscopy. **(A)** Adipogenic differentiation of ADSC (phase-contrast); **(B)** Adipogenic differentiation of ADSC (phase-contrast + fluorescence); **(C)** Adipogenic differentiation of ADSC (fluorescence).

**Figure 3:**
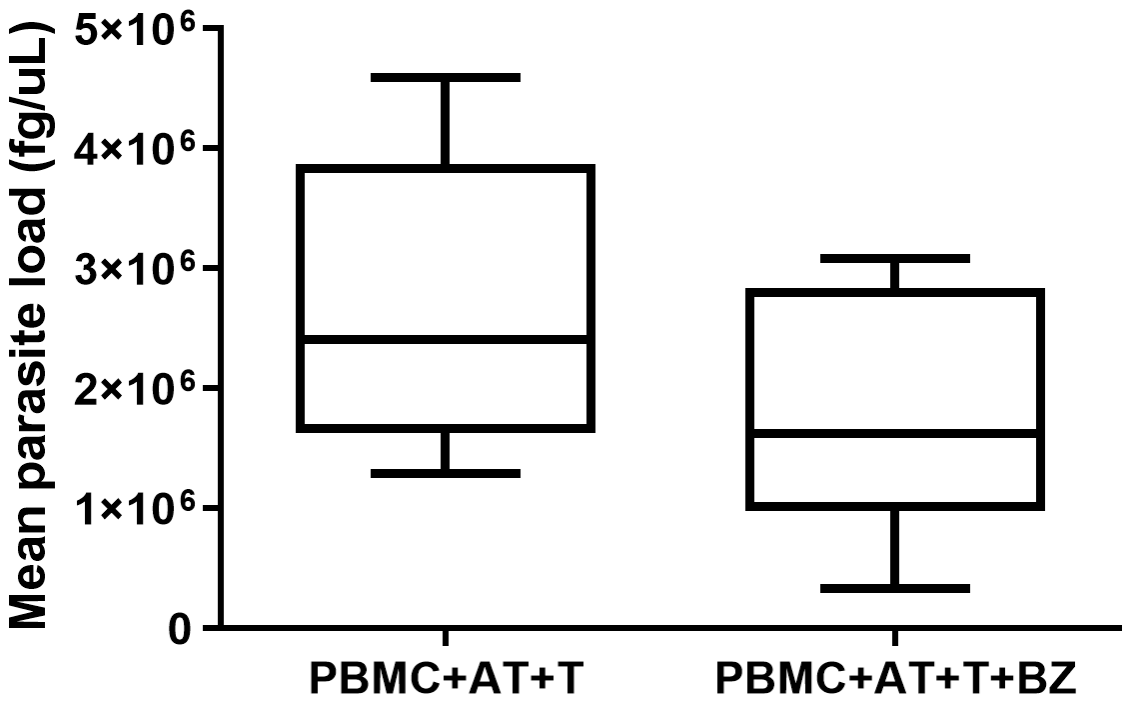
Quantification of parasite load of *Trypanosoma cruzi* infected adipocytes in indirect culture between adipose tissue, peripheral blood mononuclear cells and Benznidazole treatment. PBMC, Peripheral blood mononuclear cells (PBMC); AT, Adipose tissue from adipogenic differentiation of ADSC (AT); *Trypanosoma cruzi* (T); Benznidazole (BZ). Horizontal bars represent the median and vertical bars the lower and upper limit. Symbols (*) above the bars indicate statistical difference with p ≤ 0.05.

Twelve days after the induction of adipogenic differentiation, we observed the presence of small lipid vesicles (multilocular) within the adipocytes (**Figure 2**). In general, there was a high parasite load in the culture conditions where the cells were infected with *T. cruzi* (**Figure 3**). However, we found that the average parasite load quantification of the PBMC+AT+T+BZ group was lower than the PBMC+AT+T, although it did not represent a statistically significant difference (p= 0.2337) (**Figure 3**).

### AT induces upregulation of IL-2, IL-6 and MCP-1 but promotes downregulation of TNF and Il-8 in the presence of PBMC and *T. cruzi*

**Figure 4:**
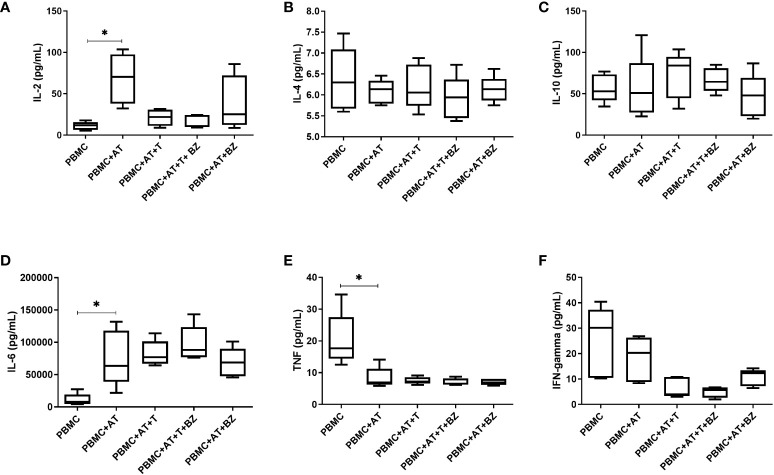
Measurement of cytokines in the culture supernatant of indirect culture between Trypanosoma cruzi-infected adipose tissue, peripheral blood mononuclear cells and benznidazole treatment. PBMC, Peripheral blood mononuclear cells (PBMC); AT, Adipose tissue from adipogenic differentiation of ADSC (AT); Trypanosoma cruzi (T); Benznidazole (BZ). **(A)** IL-2 cytokine measurement; **(B)** IL-4 cytokine measurement; **(C)** IL-10 cytokine measurement; **(D)** IL-6 cytokine measurement; **(E)** TNF cytokine measurement; **(F)** IFN-gamma cytokine measurement. Horizontal bars represent the median and vertical bars the lower and upper limit. Symbols (*) above the bars indicate statistical difference with p ≤ 0.05.

Regarding cytokines, it was possible to observe that the presence of AT (PBMC+AT), even if indirectly, promotes an increase in IL-2 production that is statistically significant (p=0.0014) compared to the PBMC condition (**Figure 4A**). On the other hand, the presence of *T. cruzi* in PBMC+AT+T and PBMC+AT+T+BZ culture conditions considerably decreased the production of this cytokine compared to the PBMC + AT condition (**Figure 4A**). The production of IL-4 and IL-10 was similar in all culture conditions evaluated (**Figures 4B, C**).

In the production of IL-6, we observed that AT promoted an increase in the inflammatory cytokine in all groups with AT when compared to the PBMC condition, thus being statistically significant in the PBMC+AT group (p=0.0172) (**Figure 4D**). We detected that TNF production decreased in all groups in which AT was present, being statistically significant (p=0.0332) between PBMC+AT when compared to the PBMC group (**Figure 4E**). However, in the presence of *T. cruzi* (PBMC+AT+T and PBMC+AT+T+BZ), there was a decrease in IFN-γ compared to the other culture conditions studied (**Figure 4F**). Yet, the production of cytokines between the infected and BZ-treated culture condition (PBMC+AT+T+BZ) was similar when compared to the only infected one (PBMC+AT+T) (**Figure 4F**).

**Figure 5:**
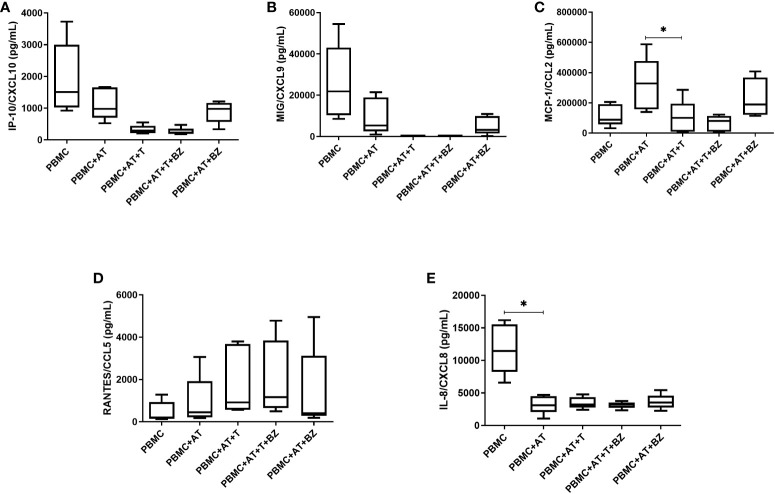
Measurement of chemokines in the culture supernatant of indirect culture between adipose tissue infected by *Trypanosoma cruzi*, peripheral blood mononuclear cells and treatment with benznidazole. PBMC, Peripheral blood mononuclear cells (PBMC); AT, Adipose tissue from adipogenic differentiation of ADSC (AT); *Trypanosoma cruzi* (T); Benznidazole (BZ). **(A)** IP-10/CXCL10 chemokine measurement; **(B)** MIG/CXCL9 chemokine measurement; **(C)** MCP-1/CCL2 chemokine measurement; **(D)** RANTES/CCL5 chemokine measurement; **(E)** IL-8/CXCL8 chemokine measurement. Horizontal bars represent the median and vertical bars the lower and upper limit. Symbols (*) above the bars indicate statistical difference with p ≤ 0.05.

As for chemokines, we found that there was a decrease in IP-10/CXCL10 and MIG/CXCL-9 in the culture conditions infected by *T. cruzi* (PBMC+AT+T and PBMC+AT+T+BZ) in comparison to uninfected controls (PBMC, PBMC+AT and PBMC+AT+BZ) (**Figures 5A, B**). Another chemokine that followed the same behavior was MCP-1/CCL2, where there is a decrease of this chemokine in conditions where *T. cruzi* is present, compared to the PBMC+AT condition, and therefore being statistically significant in the PBMC+AT+T group (p=0.0106) (**Figure 5C**).

For RANTES/CCL-5, we found that the averages of the culture conditions studied were similar to each other, except in the culture conditions infected by *T. cruzi* (PBMC+AT+T and PBMC+AT+T+BZ), where there was a higher production of this chemokine (**Figure 5D**). In contrast, for IL-8/CXCL-8, we observed that AT promotes an inhibition/decrease in all culture conditions compared to PBMC and is statistically significant for the PBMC+AT condition (p=0.0208) (**Figure 5E**). It is worth noting that this same phenomenon occurred with the cytokine TNF (**Figure 4D**).

### BZ decreases adipsin in AT infected with *T. cruzi* in the presence of PBMC

**Figure 6:**
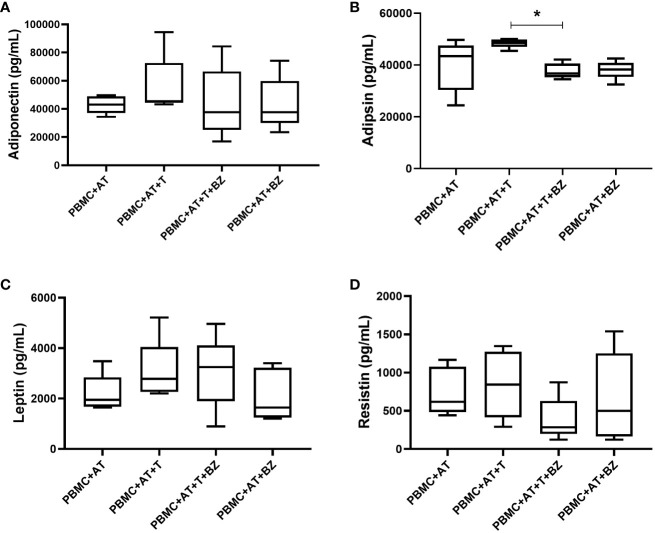
Measurement of adipokines in the culture supernatant of indirect culture between adipose tissue infected by *Trypanosoma cruzi*, peripheral blood mononuclear cells and treatment with benznidazole. PBMC, Peripheral blood mononuclear cells (PBMC); AT, Adipose tissue from adipogenic differentiation of ADSC (AT); *Trypanosoma cruzi* (T); Benznidazole (BZ). **(A)** Adiponectin adipokine measurement; **(B)** Adipsin adipokine measurement; **(C)** Leptin adipokine measurement; **(D)** Resistin adipokine measurement. Horizontal bars represent the median and vertical bars the lower and upper limit. Symbols (*) above the bars indicate statistical difference with p ≤ 0.05.

Regarding adipokines, there was high production of adiponectin, leptin and resistin, which is similarly distributed among the culture conditions studied (**Figures 6A, C, D**). However, although adipsin is widely secreted in the culture conditions studied, we observed that there was a greater increase in the PBMC+AT+T condition in contrast with the treated culture condition (PBMC+AT+T+BZ), then being statistically significant (p=0.0207) (**Figure 6B**).

### AT infected by *T. cruzi* promotes decreased expression of CD80^+^ and HLA-DR^+^ molecules in CD14^+^ cells

**Figure 7:**
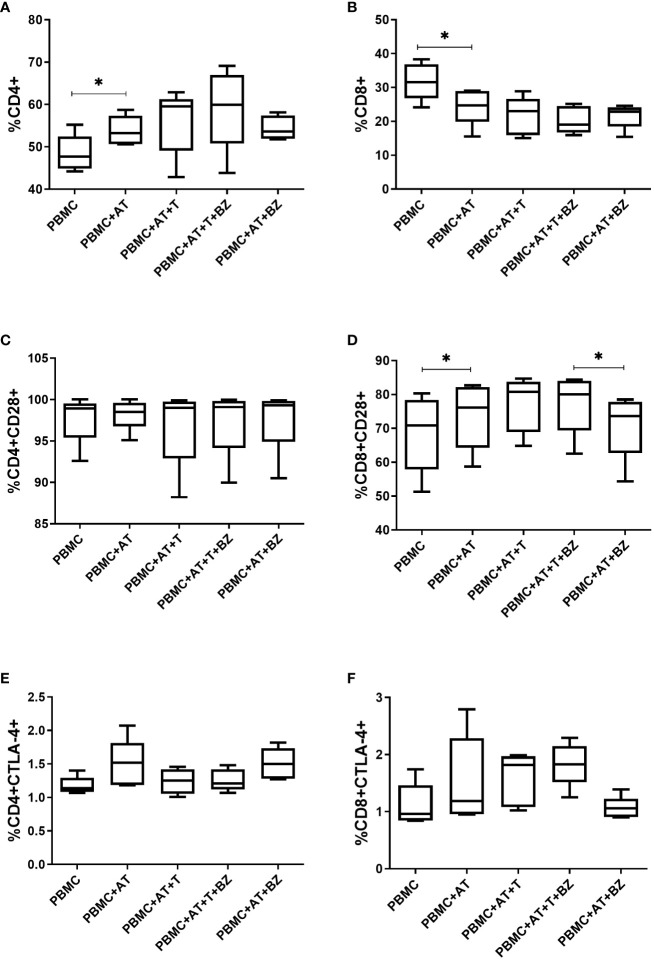
Evaluation of the expression of activation/inhibition molecules in CD4+ and CD8+ T lymphocytes from indirect culture between Trypanosoma cruzi-infected adipose tissue, peripheral blood mononuclear cells and benznidazole treatment. PBMC, Peripheral blood mononuclear cells (PBMC); AT, Adipose tissue from adipogenic differentiation of ADSC (AT); Trypanosoma cruzi (T); Benznidazole (BZ). **(A)** CD4+ expression on lymphocytes; **(B)** CD8+ expression on lymphocytes; **(C)** CD28+ expression on CD4+ T lymphocytes; **(D)** CD28+ expression on CD8+ T lymphocytes; **(E)** CTLA-4+ expression in CD4+ T lymphocytes; **(F)** CTLA-4+ expression in CD8+ T lymphocytes. Horizontal bars represent the median and vertical bars the lower and upper limit. Symbols (*) above the bars indicate statistical difference with p ≤ 0.05.

Regarding the frequency of the lymphocyte population present in the indirect culture, we found that there was a higher frequency of CD4^+^ T lymphocytes in the culture conditions in which there is the presence of AT, being statistically significant in the PBMC+AT condition (p=0.0025) when compared to PBMC (**Figure 7A**). In contrast, the opposite behavior in CD8^+^ T lymphocytes was noticed, in which the presence of AT decreases the frequency of these cells, also having statistical significance in the PBMC+AT condition (p=0.0037) compared to PBMC (**Figure 7B**).

When evaluating the surface molecules responsible for cell activation and inhibition, we observed that the frequency of CD28^+^ molecules in CD4^+^ T lymphocytes was similar in all culture conditions studied (**Figure 7C**). Still, in CD8^+^ T lymphocytes, there was a higher frequency of CD28^+^ in culture conditions where there is the presence of AT, which was found in PBMC+AT in contrast with the PBMC group (p=0.0141), as well as in PBMC+AT+T+BZ when compared to PBMC+AT+BZ (p=0.0222) (**Figure 7D**).

Moreover, the research concluded that the frequency of CTLA-4 in CD4^+^ T lymphocytes was similar between culture conditions, except for the PBMC+AT and PBMC+AT+BZ groups that showed higher medians regarding the frequency of this molecule in comparison with the other culture conditions (**Figure 7E**). However, in CD8^+^ T lymphocytes, according to the average of each group, we observed that in the conditions in which the cells were infected (PBMC+AT+T and PBMC+AT+T+BZ), they demonstrated a higher frequency of CTLA-4 than the other culture conditions (**Figure 7F**).

**Figure 8:**
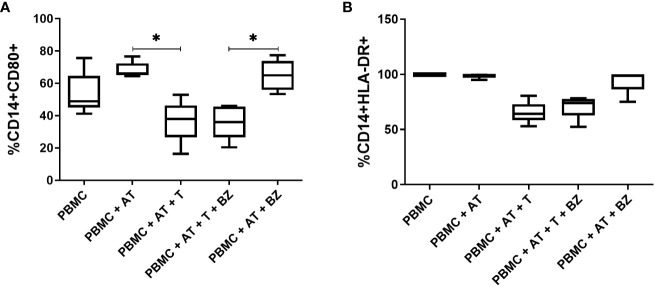
Evaluation of the expression of activation/inhibition molecules in CD14^+^ cells from indirect culture between *Trypanosoma cruzi*-infected adipose tissue, peripheral blood mononuclear cells and benznidazole treatment. PBMC, Peripheral blood mononuclear cells (PBMC); AT, Adipose tissue from adipogenic differentiation of ADSC (AT); *Trypanosoma cruz*i (T); Benznidazole (BZ). **(A)** CD80+ expression on CD14+ monocytes; **(B)** HLA-DR+ expression on CD14+ monocytes Horizontal bars represent the median and vertical bars the lower and upper limit. Symbols (*) above the bars indicate statistical difference with p ≤ 0.05.

Regarding CD14^+^ cells, it was ascertained that the frequency of CD80^+^ was higher in the PBMC+AT group compared to PBMC+AT+T (p=0.0114), as well as in the PBMC+AT+BZ group compared to PBMC+AT+T+BZ (0.0008) (**Figure 8A**). However, in CD14^+^HLA-DR^+^ cells, there was a decrease in the frequency of this cell population in the culture conditions infected by *T. cruzi* (PBMC+AT+T and PBMC+AT+T+BZ), when compared to the other groups (**Figure 8B**).

## Discussion

AT has, for many years, been considered only an energy backup (22). Regardless, this endocrine organ plays a crucial role in the immune response, such as secreting adipokines and cytokines and acting in the host’s energy homeostasis (9, 23). Also, the AT has been seen as a “sanctuary” for several microorganisms – such as viruses, bacteria and parasites – that use this mechanism to try to escape the host’s immune response (24, 25). Therefore, our object of study was the AT as a reservoir for *T. cruzi* (7). However, as the human host is complex and the AT is a very heterogeneous organ, composed of several cell types (26), we mimicked an *in vitro* microenvironment in which there is indirect contact of the AT infected by *T. cruzi* and the PBMC in addition to verifying if there is any repercussion in the face of treatment with BZ. As a result, we found that indirect contact between PBMC and infected AT is sufficient to promote immunomodulatory changes that can stimulate the production of cytokines such as IL-6 and IL-2 and reduce the production of TNF and IL-8/CXCL8.

During our study, we used ADSC to perform adipogenic differentiation, which, although possessing AT as its source, can differentiate other cell types according to the stimulus to which it is applied (27). Thus, we observed that after the differentiation induction, the differentiated cells’ lipid droplets were multilocular, which may be associated with BAT or beige adipocytes. Although our differentiation kit does not define which type of AT will be differentiated, we believe that adipogenic inducers have a determining role in cell type specialization because, depending on the concentration of the compounds, the AT can differentiate into distinct subtypes (28).

BAT and beige adipocytes are associated with thermogenesis processes. However, they have different origins (29). Beige adipocytes derive from white adipocyte precursors and are found in scattered clusters within WAT (30). On the other hand, BAT occurs separately in deposits more associated with the cervical, axillary and interscapular regions (8). In agreement with our study, Rashnonejad et al. (31) have already found that ADSC and umbilical cord-derived stem cells present adipocytes with unilocular lipid droplets – when differentiated into WAT – and multilocular lipid droplets – when differentiated into BAT. Although this was not the aim of this study, phenotypic markers could assist in this classification of AT type.

*In vitro* treatment with BZ did not statistically alter the production of cytokines, chemokines and the quantification of *T. cruzi* DNA under the culture conditions in which the cells were infected in a significant way. Even so, *in vitro* studies using PBMC from chronic patients with Chagas disease or volunteers who were infected with *T. cruzi* were able to reduce the exacerbated inflammation generated by the infection through the administration of BZ (32, 33). In addition, our research group – also using an *in vitro* model – found that AT infected with *T. cruzi* and treated with BZ showed a reduction in parasite load and inflammation through a decrease in IL-6, when compared to the AT that was only infected (20). However, according to the results derived from our study, the use of AT as an escape from the immune response may be an alternative to the parasite infection since the efficiency of the drug may be reduced. Furthermore, interaction with PBMC significantly altered immune modulation and the effect of BZ on infected AT, strengthening the hypothesis that these cells can affect the immunometabolic balance even when acting indirectly.

Regarding immunomodulation, it could be concluded that the interaction between AT and PBMC promotes the production of the cytokine IL-2 when compared to the other groups. IL-2 plays a central role in regulating immune cells’ proliferation, activation and homeostasis (34–36). This cytokine is mainly secreted into the circulation by activated CD4^+^ and CD8^+^ T cells and, to a lesser extent, by dendritic cells and macrophages (36). In the study by Kochumon et al. (37), it was observed that IL-2 expression was upregulated in obese subjects compared to lean subjects. Furthermore, this elevation of IL-2 is associated with insulin resistance and several inflammatory markers. Thus, the results of Kochumon et al. (37) corroborate our study since adipogenic differentiation promotes fat accumulation within cells, which leads to an inflammatory state.

Similarly, we found that, in culture conditions where AT is present, IL-6 production occurs regardless of *T. cruzi* infection. Our data corroborates the literature since IL-6 belongs to the group of adipokines. These bioactive factors are secreted by WAT but can also be produced by cells that infiltrate AT, such as macrophages (38). Yet, curiously enough, *T. cruzi* infection did not promote a significant increase in IL-6 compared to the PBMC+AT condition. In contrast, Moreira et al. (20) noticed that treatment with BZ in *T. cruzi*-infected AT led to a decrease in IL-6 compared to untreated AT. Nonetheless, in this case, there was no influence of PBMCs in the microenvironment.

In the study held by Gonzaléz et al. (39) investigating the cytokine profile, an increase in IL-6 and TNF was observed in chronic chagasic patients. However, compared to healthy individuals, these patients had metabolic imbalances such as high body mass index (BMI) and high blood glucose and insulin resistance. Moreover, Wueest and Konrad (40) have already highlighted that IL-6 signaling in adipocytes is involved in the development of insulin resistance associated with obesity and hepatic steatosis by inducing the release of free fatty acids and leptin from adipocytes, thus affecting hepatic metabolism and pancreatic β-cell function. Therefore, not only does the infection promote inflammatory microenvironment – fatty acid synthesis *in vitro* model – but also obesity and metabolic dysfunctions act on IL-6 elevation (41–44).

In opposition, TNF secretion remained at basal levels in the presence of AT. TNF, like IL-6, is also considered an adipokine since it is well-established that isolated adipocytes and those differentiated from other cell populations can produce TNF (45). In spite of that, adipose tissue also contains a stromal vascular fraction (SVF), possessing several metabolically relevant cell types. These include preadipocytes, endothelial cells, smooth muscle cells, fibroblasts, leukocytes and macrophages. More recent studies have even shown that SVF can produce substantially more TNF than adipocytes (46–49).

Combs et al. (10) previously observed that visceral AT from mice infected with the Brazil strain of *T. cruzi* showed elevated TNF, IFN-γ and IL-1 and reduced adiponectin and leptin. Elevation of the pro-inflammatory cytokines TNF, IFN-γ and IL-1β has also been demonstrated in adipocytes differentiated from the murine fibroblasts infected by *T. cruzi* (11). Yet, some factors may have contributed to the decreased TNF levels in our indirect culture. These include adipogenic inducers, most notably indomethacin, a PPAR-γ associated with decreased TNF when administered to human monocytes (50). PPAR-γ is an essential regulator for adipogenesis and is highly necessary for maintaining the mature adipocyte phenotype (51). Even the decrease of PPAR-γ in mature adipocytes compromises their viability and fat storage because there is a loss of expression of key metabolic enzymes (52, 53). Several agents that promote the differentiation of fibroblast lineages into adipocytes are PPAR-γ agonists, including prostaglandins, oral antiglycemics and various non-steroidal anti-inflammatory drugs (50).

Additionally, in the study held by González et al. (54), it was shown that adipocytes and SVF collected from mice show decreased PPAR-γ in infected mice when compared to uninfected controls and that this decrease is correlated with TNF elevation. In studies with no *T. cruzi* infection, we can also verify a decrease in TNF in obese individuals after bariatric surgery correlated with the loss of fat and weight (55, 56).

In correspondence with our findings, Fain et al. (46) found that TNF production by human visceral and subcutaneous adipose tissue explants occurs between 4 and 48 hours of culture incubation, suggesting that TNF is a short-lived cytokine in primary culture. Also, more than 95% of the cytokine secretion was related to non-adipose cells. During another study from our group, we observed that *T. cruzi* infection in PBMC already induces TNF production within 24 hours after culture initiation and significantly decays between 5 and 10 days (32). Therefore, as our culture was performed 72 hours after BZ treatment, the peak of TNF production may have already decayed to basal levels.

Regarding IL-10, although cytokine production occurs, it does so similarly between culture conditions. Both myeloid and lymphoid cells can secrete IL-10 depending on the stimulus applied. These cells include macrophages, monocytes, dendritic cells, neutrophils, mast cells, eosinophils, natural killer cells, CD4+, CD8+ T cells, and B cells (57). In addition, some non-hematopoietic cells, including epithelial cells, are also capable of producing IL-10 (58). So, we believe IL-10 secretion among culture conditions is associated with cultured PBMC.

On another note, *T. cruzi* infection promotes a decrease in the chemokines IP-10/CXCL10 and MIG/CXCL9 in the supernatant content of the culture. The chemokine IP-10/CXCL10 is activated by binding to the CXCR3 receptor, typically expressed on T, B, natural killer (NK) cells, dendritic cells and macrophages. Abnormal levels of IP-10/CXCL10 have been identified in the body fluids of individuals infected with viruses, bacteria, fungi and parasites (59–62). Similarly, MIG/CXCL9 binds to the CXCR3 receptor (63).

In the study done by Kiran et al. (64), CD8^+^ T cells and AT from the epididymis of mice subjected to a high-fat diet showed elevation of IP-10/CXCL-10 and MCP-1/CCL2. Moreover, there is an increased expression of CXCR3 receptors on CD8+ T cells in contact with AT. However, AT also expresses CXCR3 receptors and modulates obesity-induced inflammation (65). For that reason, we suggest that in addition to the high expression of receptors caused by contact between AT and PBMC the infection enhances this increase, which may escalate the consumption of these chemokines in this microenvironment.

The chemokine MCP-1/CCL2 was widely secreted in all culture conditions, but the PBMC+AT interaction obtained the highest amount. The primary sources of MCP-1 are epithelial cells, endothelial cells, smooth muscle cells, monocytes/macrophages, fibroblasts, astrocytes and microglial cells regulated by several other cytokines and factors (66). Still, MCP-1 is associated with several metabolic issues, such as insulin resistance and obesity (67–70). In accordance with our study, Cabalén et al. (71) found that *T. cruzi*-infected mice fed an obesity-inducing diet showed elevated MCP-1/CCL2 levels. Additionally, it has already been reported that saturated fatty acids can induce the secretion of MCP-1/CCL2 and other inflammatory adipokines in murine 3T3-L1 adipocytes (72). Therefore, as AT and PBMC secrete MCP-1/CCL2, we believe that the decreased levels of this chemokine in culture conditions in which the parasite is present are attributed to the metabolism of *T. cruzi* itself, which, when multiplying and infecting new cells, promotes a reduction in lipid content and thus reduces the production of the chemokine.

Similar to what was observed with TNF, the chemokine IL-8/CXCL8 had the same behavior in the presence of TA. IL-8/CXCL8 is a chemokine produced by nucleated cells, mainly macrophages, which are actively involved in inflammation by promoting the recruitment of monocytes and neutrophils (73). Furthermore, macrophage-derived foam cells and adipocytes have been found to express and secrete this chemokine extensively (70).

In this regard, Ballesteros et al. (74) found that AT-derived stem cells supplemented with platelet lysate showed increased IL-8 and decreased CXCR2 receptor expression. Also, the authors point out that any changes in the expression of chemokine receptors may also result from factors secreted during the culture of AT-derived stem cells. However, increased CXCR2 expression on monocytes/macrophages has already been associated with atherosclerosis and found on PBMC and within atherosclerotic plaques in humans and murine (75–77).

The interaction of adipocytes and macrophages modulates the maintenance of metabolic and immune homeostasis (78). When cultured with RAW264.7 macrophages in a Transwell system, bone marrow mesenchymal stem cells can induce macrophages to polarize to M2 phenotype and secrete anti-inflammatory and immunomodulatory cytokines (79). Macrophages residing in lean AT tissues exhibit characteristics of anti-inflammatory M2-like macrophages and have a positive role in maintaining AT homeostasis, preventing obesity-induced inflammation and thus promoting insulin sensitivity. In contrast, infiltrated macrophages, mainly M1-like macrophages, are more commonly associated with AT inflammation and insulin resistance (80). Consequently, we suggest that the interaction of AT+PBMC may promote increased expression of CXCR1/CXCR2 receptors or modulation of macrophage phenotype that contribute to the decay of the chemokine IL8/CXCL8 and other cytokines, such as TNF, which should be investigated in the future.

There was high secretion of these molecules in all culture conditions studied regarding adipokines. Yet, for adipsin, an interesting phenomenon occurred: the treatment with BZ promoted a decrease in the secretion of this adipokine in the infected AT. Adipsin, also known as complement factor D, is a serine protease secreted by adipocytes associated with forming C3 convertase by the complement system, but it also involves triacylglycerol synthesis in human adipocytes (81). Studies have emphasized that high levels of adipsin are associated with overweight/obese individuals and related to an increased cardiovascular risk in women with polycystic ovary syndrome (82, 83). In another study by Prugger et al. (84), higher plasma levels of adipsin were associated with an increased 10-year risk of ischemic stroke in a cohort of healthy middle-aged men. Although data regarding parasites and adipsin is still scarce, our findings comply with the literature since this adipokine was associated with inflammatory conditions. Also, BZ exerted a beneficial effect by reducing adipsin in infected AT.

Other adipokines, such as leptin and adiponectin, are intrinsically related to host metabolism. Leptin is mainly produced by the AT, associated with the amount of body fat stored. It is also involved in regulating food intake, neuroendocrine function, reproduction, angiogenesis and blood pressure (85). Circulating leptin levels correlate mainly with total body fat and are thus increased in obese individuals (86). Through *in vitro* experiments, the effects of leptin are associated with decreased lipogenesis, increased triglyceride hydrolysis and fatty acid oxidation (87).

Adiponectin is also secreted almost exclusively by the AT. It has cardioprotective functions, protecting against insulin resistance and excessive hepatic lipid accumulation and exerts anti-inflammatory effects (88). Circulating levels of this adipokine in obese patients are also decreased (89). Through *in vitro* experiments, adiponectin overexpression in 3T3-L1 cells increases lipid storage and adipogenesis (90).

González et al. (54) found that leptin and adiponectin expression decrease upon *T. cruzi* infection in 3T3-L1 adipocytes and AT of mice compared to uninfected mice. However, Lizardo et al. (91) observed the presence of disintegrated adipocytes with multilocular lipid droplets and infiltrated immune cells in AT 90 days post-infection. Even so, fat loss increased adiponectin and PPARγ levels, indicating that apoptotic cell death of adipocytes induces pro-adipogenic signaling (91). Therefore, leptin/adiponectin levels may vary according to the type of infection/phase of Chagas disease and metabolic imbalance (92). Given these findings, we believe that in our study, PBMC modulated adipokine secretion in infected AT.

Regarding cell activation and inhibition molecules, we noticed that the presence of AT promoted an increase in the frequency of CD4^+^ T cells. Still, the same was not observed when the frequency of activated CD4^+^ T lymphocytes was measured. In humans, CD4^+^ T lymphocytes represent the main population of lymphocytes in adipose tissue. In general, CD4^+^ T cells depending on cytokine stimulation may develop other activation states (93, 94).

On the other hand, in CD8^+^ T lymphocytes, there was a decrease in the frequency of these cells in the presence of AT. Nevertheless, a higher frequency of activated CD8+ T cells in the presence of AT or *T. cruzi* infection was also verified. Similar to our findings, in the study by Nishimura et al. (95), a higher frequency of effector CD8+ T cells infiltrating the epididymal adipose tissue of mice ingesting a high-fat diet (HFD) was observed compared to the frequency of effector CD4^+^ T cells. Furthermore, the study suggests that obese AT activates CD8^+^ T cells, promoting macrophage recruitment and activation in this tissue.

In another study, by culturing CD8^+^ T cells and adipocytes from epididymal tissue of mice subjected to HFD, it was found that monocyte-to-macrophage differentiation and phenotypic polarization was mediated by HFD-induced CD8^+^ T cell interaction with adipocytes (64). These findings were confirmed because both CD8^+^ T cells and adipocytes that did not undergo HFD when cultured alone did not induce sufficient macrophage amount for differentiation, which strengthens the hypothesis of the relationship between CD8^+^ T cells, adipocytes and inflammation (64).

Interestingly, in CD14^+^ cells, we found a decreased frequency of CD80^+^ in *T. cruzi*-infected cell culture conditions. However, in PBMC infected by *T. cruzi*, an increase in the frequency of this molecule has already been observed in culture conditions infected by the parasite (32). Accordingly, this decrease may function as a defense mechanism for *T. cruzi* to remain in the AT and escape the host immune response.

**Figure 9:**
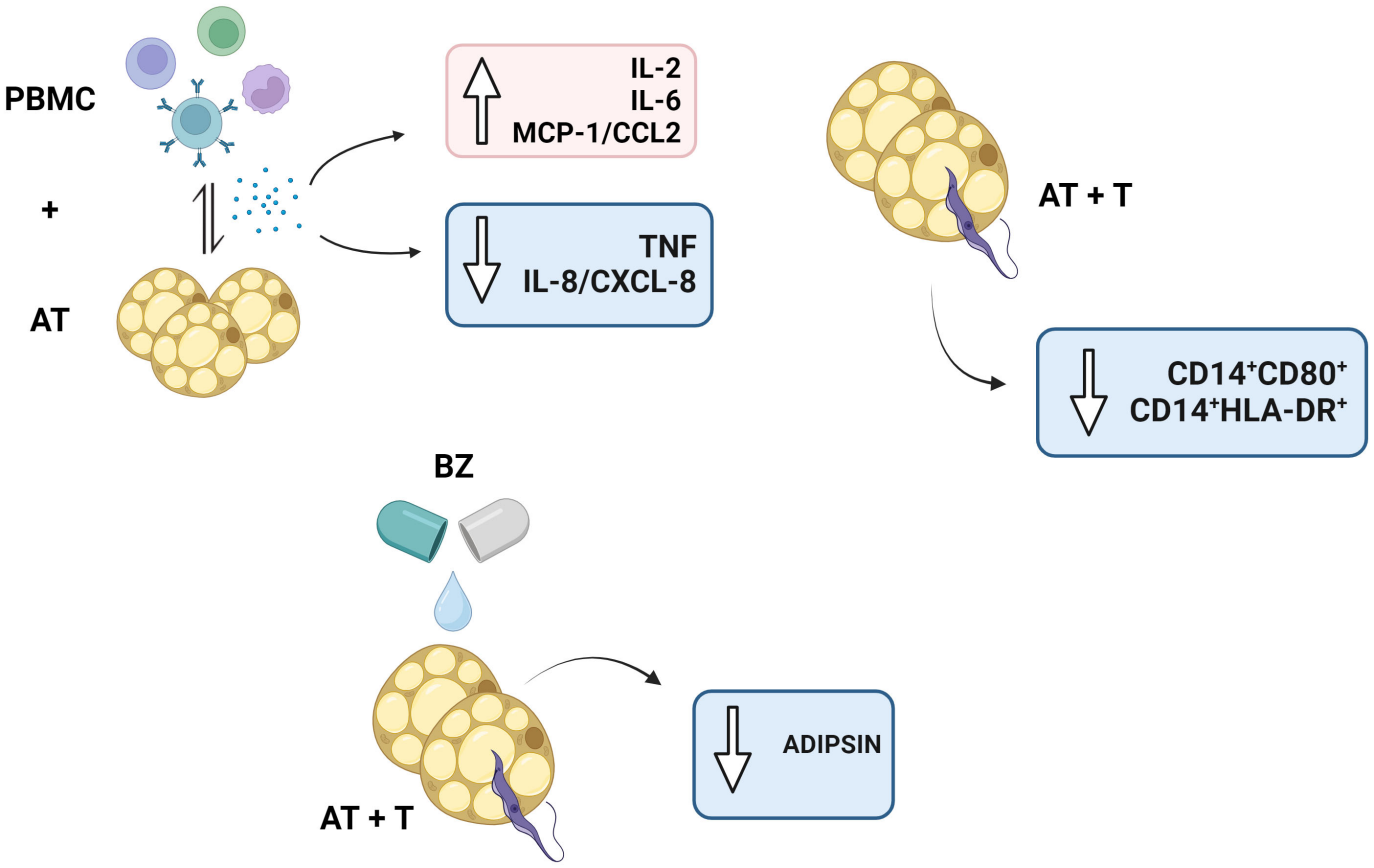
Schematic representation of the study’s main findings. PBMC, Peripheral blood mononuclear cells (PBMC); AT, Adipose tissue from adipogenic differentiation of ADSC (AT); *Trypanosoma cruzi* (T); Benznidazole (BZ). Design created with the aid of Biorender ^®^.

In conclusion, our data indicates that BZ failed to promote and induce important immunomodulatory mechanisms in the infected AT, which is beneficial for *T. cruzi* and may use the AT as an ally in escaping the host immune response. Moreover, the interaction between PBMC and AT promotes mechanisms that may affect AT immunometabolism (**Figure 9**). Besides, as this is an *in vitro* study, a drug that has not undergone enzymatic metabolization to its active form or the long-term cultivation of infected AT should be considered. As a result, studies *in vivo* models may provide more evidence of how the parasite can use AT as a defense. Withal, our indirect *in vitro* culture model may be interesting for drug studies that evaluate the efficiency of drugs in this microenvironment.

## Data availability statement

The raw data supporting the conclusions of this article will be made available by the authors, without undue reservation.

## Ethics statement

The studies involving humans were approved by Research Ethics Committee - IAM/Fiocruz (C.A.A.E 97930918.3.0000.5190). The studies were conducted in accordance with the local legislation and institutional requirements. The participants provided their written informed consent to participate in this study.

## Author contributions

LM: Conceptualization, Data curation, Formal analysis, Investigation, Methodology, Software, Visualization, Writing – original draft, Writing – review & editing. AS: Formal analysis, Investigation, Methodology, Visualization, Writing – review & editing. CC: Methodology, Writing – review & editing. CS: Methodology, Writing – review & editing. KO: Methodology, Writing – review & editing. DT: Methodology, Writing – review & editing. MB: Methodology, Writing – review & editing. MR: Formal analysis, Funding acquisition, Resources, Writing – review & editing. VL: Conceptualization, Formal analysis, Funding acquisition, Project administration, Resources, Supervision, Validation, Writing – review & editing.

## Supplementary material

Supplementary Figure 1 Acquisition strategy of adipokines dosage in the culture supernatant of the indirect culture between adipose tissue infected by *Trypanosoma cruzi*, peripheral blood mononuclear cells and benznidazole treatment.
