# Supplementary figures and images for: Interaction between peripheral blood mononuclear cells and Trypanosoma cruzi-infected adipocytes: implications for treatment failure and induction of immunomodulatory mechanisms in adipose tissue
Source: Front Immunol. 2024 Mar 12;15:1280877. doi: 10.3389/fimmu.2024.1280877 (PMC10963431; doi:10.3389/fimmu.2024.1280877)

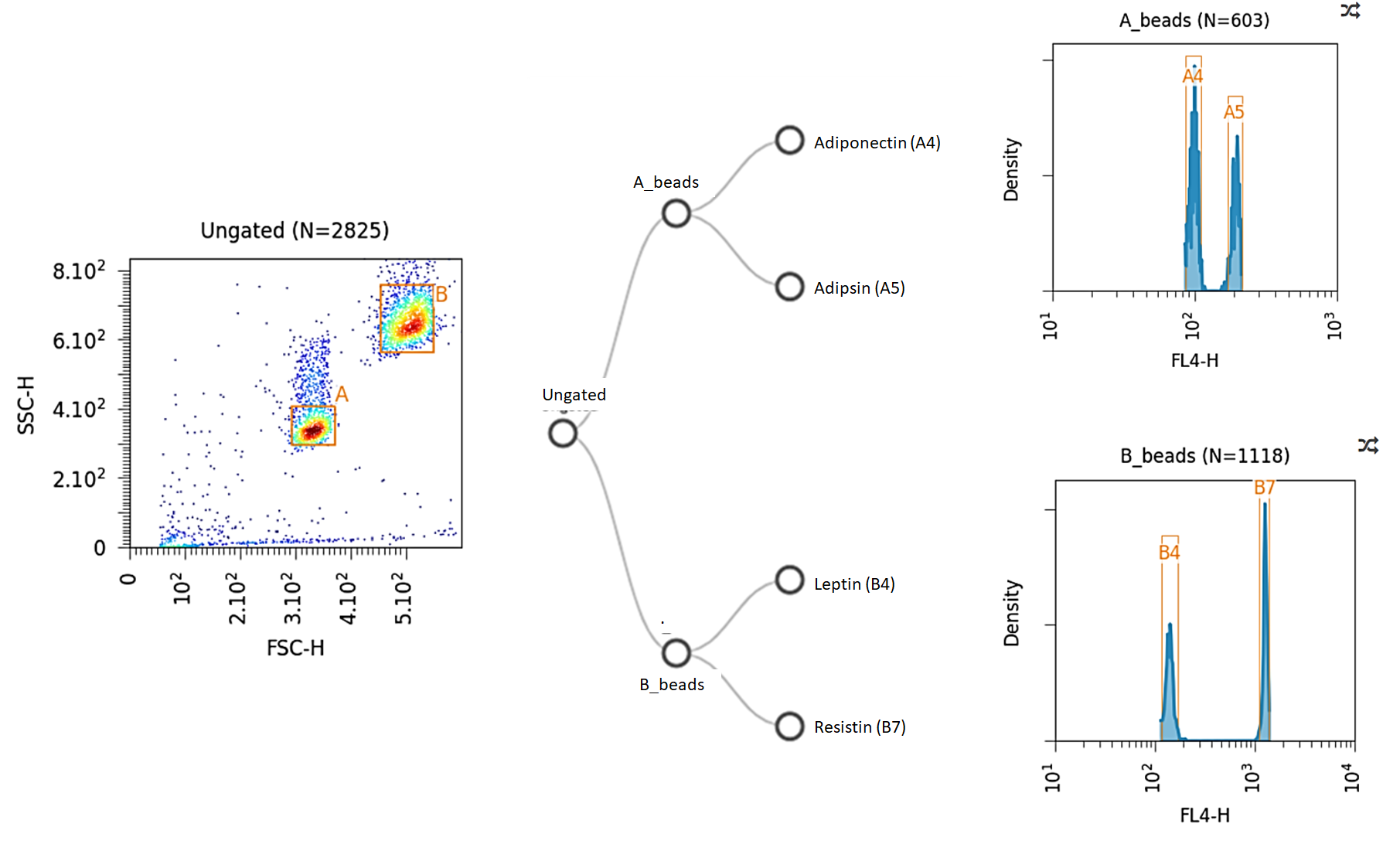

Supplement: Supplementary Figure 1 — Acquisition strategy of adipokines dosage in the culture supernatant of the indirect culture between adipose tissue infected by Trypanosoma cruzi, peripheral blood mononuclear cells and benznidazole treatment. [file Image_1.jpeg]
